# Moving From Representativeness Toward Transportability in an Era of Digital and Big Data

**DOI:** 10.3389/ijph.2026.1609306

**Published:** 2026-03-27

**Authors:** Arnaud Chiolero

**Affiliations:** 1 Population Health Laboratory (#PopHealthLab), University of Fribourg, Fribourg, Switzerland; 2 Swiss School of Public Health (SSPH+), Zurich, Switzerland; 3 School of Population and Global Health, McGill University, Montreal, QC, Canada

**Keywords:** data science, epidemiology, evidence, population health, representativeness

## Abstract

Evidence-based public health demands that study findings provide meaningful insights into improving the health of target populations, making representativeness a widely regarded hallmark of high-quality epidemiological research. However, big data and the digital health datademic are changing the way target and study populations are defined and how to ensure the external validity of study findings. What matters is assessing the degree of transportability of these findings—how well they inform about the target population. I review the gain of shifting the focus away from study representativeness and instead prioritizing the explicit assessment and reporting of transportability.

## Introduction

Evidence-based public health demands that study findings provide meaningful insights into improving the health of target populations, making representativeness a hallmark of high-quality epidemiological study [1, 2]. Representativeness is, however, neither necessary, nor sufficient to guarantee the external validity of study findings [1]. Further, big data and the digital health datademic—a concept related to infodemic to describe the overabundance of data [3] and the challenge of using them to produce actionable information—are changing the way target and study populations are defined and how to ensure the external validity of study findings [4]. While minimizing selection bias and ensuring similarity between a study sample and its target population help, they are not sufficient to guarantee external validity. What matters is assessing the degree of transportability of study findings, i.e., how well these findings inform about the target population—the population on which information is searched to assess its health status and disease burden, as well as their causes, and to guide health related decisions [5, 6]. In this education article, I review the gain of shifting the focus away from study representativeness and instead prioritizing the explicit assessment and reporting of transportability.

## The End of Representativeness

According to the Cambridge dictionary, representativeness is defined as “the fact of a smaller group of people or things representing a larger group accurately, so that the smaller group is typical of the larger one” [7]. Without a census, epidemiologists and other population health scientists typically consider a study representative if it is conducted on a random sample of the target population [8]. In this context, if the researchers are lucky enough with the sampling, the study’s descriptive or causal estimate—once internal validity biases due, e.g., to measurement errors and confounding are accounted for—provides insight into the true value of the descriptive or causal estimand in the target population, with a known degree of uncertainty. The notion of estimand is meant here to describe the target quantity, i.e., the goal of a descriptive or causal assessment in a target population, while the estimate is what is obtained from a given analysis in a specific study population [9].

However, while random sampling is one approach to achieving representativeness, it is neither necessary nor sufficient to ensure that study findings will apply to a target population [1]. Further, a growing number of epidemiological and population health studies rely on non-random samples that neither represent the reference population—the group from which study participants were drawn—nor the target population relevant for policymaking [4, 10]. This is particularly evident in studies using various types of real-world or big data, which include vast numbers of individuals who do not originate from a well-defined reference population, do not represent the target population of interest, and whose selection process (beyond self-selection) cannot be fully identified.

For instance, many studies using digital-trace data—from smartphones and wearables, search engines, social media, or streaming platforms—cannot identify the populations that generate these data (Box 1) [11]. Beyond basic sociodemographics, high-quality information on users’ characteristics is often lacking, limiting stratified analyses. Access is also constrained by the companies that hold these data. Digital data donation—users sharing the data held by these companies with researchers [12]—can reveal the data-generation process needed to assess transportability, but such donations must be widespread to yield meaningful, population-level insights.

BOX 1 **Digital traces to assess cancer trends.**

Assessing cancer trends is a core public health task, typically relying on population-based registries that collect high-quality incidence and mortality data for a defined geographic population. When exhaustive, registries are representative, but they require substantial resources and often lack timeliness. Could web queries help? Search volumes for cancer-related terms have been shown to correlate with registry incidence [13, 14]. However, such correlations provide limited—if any—transportable, actionable, population-level information. First, query volumes do not yield incidence estimates; they may reflect public concern rather than true disease burden. Second, users of web services may not represent the target population. Third, reproducibility over time is poor because the composition of users and search-engine designs change unpredictably [15].

**FIGURE 1:**
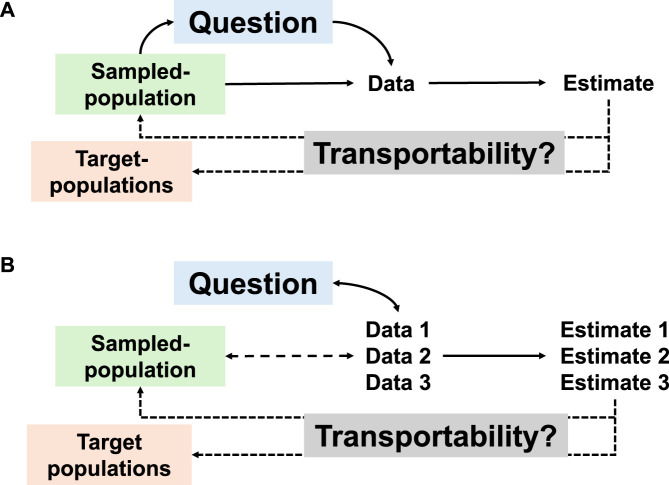
Relationship between the sampled and target populations, the descriptive or causal question (the estimand), the data collected, and the estimates. **(A)** standard epidemiological study; **(B)** epidemiological study using real-world and big-data (#PopHealthLab, 2026).

Without a well-defined reference population, the population coverage remains unknown [7], leaving these real-world-, digital-, and big-data as little more than a shifting and misrepresentative mix of multiple, often changing, populations [4, 16]. Unstable or poorly documented study populations contributes to the poor reproducibility of epidemiological research findings [17]. Using this type of data, increasingly accessible in a digital health era, is changing radically the way we define study and target populations, and the way we shape an epidemiological research question (the estimand). Hence, as shown in the Figure 1, in a classical epidemiological study (Panel A), the question (estimand) is used to define the data collected, allowing the production of an estimate. In an era of big data and datademic (Panel B), the question is shaped by the available data, in an iterative process. Data are generated by the population, but they can also define the population [4], and multiple estimates are produced.

Of note, even when a study is conducted in a random sample of a well-defined target population, following a proper sampling frame and plan, declining participation rates increase selection bias and threaten representativeness [18]. For example, the impact of the low participating rate on the representativeness of the famous UK biobank is well known, but its effect on the transportability of findings might have been underappreciated (Box 2) [19, 20]. Ultimately, representativeness is like a benevolent spirit which is haunting studies—often longed for, sometimes desperately [2], yet increasingly elusive in the era of big data [8]. It is timely to reassess representativeness and external validity through the lens of transportability [5, 6, 21].

BOX 2 **Representativeness of UK biobank and transportability of study findings.**

The UK Biobank, a large-scale cohort of over 500,000 participants, has generated major advances across medicine, epidemiology, genetics, and social science [22]. Only 5.5% of the nine million invited individuals participated, producing a non-representative volunteer sample with healthier lifestyles, higher education, and better health than the general UK population [20]. While such selection is viewed as problematic for estimating prevalences, it can also bias exposure–outcome effect estimates. To correct volunteer bias and assess to which extent study findings were transportable, researchers constructed inverse-probability weights using a representative sample of the UK Biobank’s target population and applied them to bivariate demographic associations [19]. Nearly all associations were biased; for several variables the bias was large, including weighted estimates having the opposite sign compared to unweighted estimates. Another study reported substantial differences between unweighted and weighted (i.e., “transported” to the target population) results for genetic correlations and Mendelian randomization estimates of socio-behavioral traits [20].

## Population Health Monitoring Versus Causal Inference

The major tasks of population health data science are description and causal inference [23], both of which are subject to internal and external validity biases, challenging transportability [6]. The role of representativeness differs, however, between these two tasks. In population health monitoring, where the goal is descriptive—such as estimating disease burden in a target population—representativeness is often necessary or at least expected. In contrast, for causal inference studies, which aim to estimate the effect of an exposure or intervention on an outcome, a representative random sample is generally not considered critical for the transportability to study findings [1].

The differences in representativeness requirements between descriptive population health monitoring and causal inference studies are, however, debatable. What truly matters is determining the extent to which study findings can be transported to the target population, particularly by accounting for the presence and distribution of modifiers of the estimand, being descriptive or causal [5, 6, 9]. Regardless of the data production process, a consequential epidemiology approach calls for explicitly reporting the transportability of study findings [24]. This is essential for health decision-makers [25], who need to assess whether observational or experimental study results are reliably extended to specific target populations, the ones that they are taking care of (Box 3) [26].

BOX 3 **Bid data paradox – what is transportable?.**

Surveys are essential tools for monitoring population opinions and health behaviors [27]. Social-media–based surveys can reach large numbers quickly. In early 2021, a representative sample of US Facebook users provided 250′000 responses per week on COVID-19 vaccination, but overestimated uptake by 17 percentage points relative to the CDC benchmark [28]. This huge sample produced a small margin of error, creating a false sense of certainty—the “big data paradox”: large datasets can increase confidence in estimates whatever the degree of bias [28]. By contrast, an online panel of ci. 1′000 weekly responses that followed survey best practices produced estimates close to the CDC benchmark. What was transportable from the Facebook survey? While such method is praised to assess differences across times or areas [11], this survey poorly tracked changes over time and showed weak concordance with the benchmark in between-state vaccination rankings [28]. These examples illustrate the importance of considering the survey “total error” framework, with transportability improved not by increasing sample size alone, but by minimizing non-sampling errors due to coverage, measurement, and selection biases.

## What Is Transportable?

Of note, it is frequent to distinguish generalizability from transportability, with generalizability being used to refer to what extent study findings can be applied to the population from which data were collected (the sampled population, see Figure 1) and transportability to what extent findings inform about other potential target populations [8, 25]. Assessing generalizability can be, however, considered as a specific case of transportability assessment, where the target population is the population from which data were collected. In this educational paper, we use transportability to refer to which extend study findings inform about any prespecified target populations, including the population from which data were collected.

On the one hand, ideally, study findings are transportable when the quantitative estimates obtained from the study sample can be generalized to the target population, that is, when the unit-specific quantity of interest can be reliably aggregated over this population [9]. On the other hand, transportability is often based on vague qualitative findings that are only tentatively extended to the target population [8, 29]. While this distinction between these two types of transportability is common, it should not be seen as a fundamental difference. Rather, it reflects varying degrees of confidence in how much—or how far—we can extend descriptive or causal estimates to the target population.

Within that logic, it is useful to apprehend transportability over a continuum [5]. In some studies, transportability is high, meaning the estimate can be confidently applied quantitatively to the target population of interest for descriptive or causal statements. In others, the transportability is low, allowing merely a rough qualitative estimation in the target population. Low transportability reveals a high level of uncertainty and might underscore the need for further research to generate estimates that are more transportable [30].

## Make Study Findings Transportable

Strengthening transportability requires accounting for at the study design stage and quantifying it during analysis. Whatever the study design, it is necessary to identify and measure modifiers of the descriptive or causal estimand [5, 9]. Transportability to the population from which data were collected can be strengthened through random sampling of this population. When random sampling is not feasible, alternative approaches such as purposive sampling—where individuals are selected based on expected heterogeneity—or stratified selection based on key modifiers can be used [5]. Randomly sampled study participants from a given population is, however, not enough to ensure transportability of study findings to other target populations [21]. Like with real-world data, information on modifiers of the descriptive or causal estimand will be necessary.

At the analytic stage, transportability is addressed through methods such as matching, outcome regression, standardization, or weighting, with the goal of adjusting the descriptive or causal estimates for different distributions of modifiers in the study sample and the target population [6, 16, 31–33]. Methods based on weighting are popular (see also Box 2) [19, 32, 33]. Calculating these weights requires a clear understanding of the selection and recruitment process, which can vary significantly, for example, between a study based on a random sample of a well-defined population and one relying on online survey participants without probability sampling (see Box 3) [3]. In the latter case, multiple assumptions are needed to approximate the population from which study participants originate, and these assumptions have to be explicitly formulated for estimating the degree of transportability to a target population.

The degree of transportability can be evaluated by assessing the similarity between the study and target populations, e.g., through a generalization score, the differences in standardized mean differences for modifiers, or a propensity score for selection if individual-level data are available for both study and target populations [5]. The key is to assess differences in the distribution of modifiers between the study and target populations and account for them in the analysis [19].

### Conclusion

For the practice of evidence-based public health, and in an era of big data and digital health, assessing transportability of study findings is more critical for population-level insights than ensuring representativeness of the study sample. In many cases, representativeness does not guarantee generalizability beyond the sampled population unless one assumes the absence of descriptive or causal modifiers. Study findings have no transportability *per se*; what matters for population health scientists is to estimate the extent to which their study findings are transportable to target populations. Whatever the study samples, the key to transportability is explicitly defining the target population that is eventually informed by the study findings.

## References

[1] Rothman KJ Gallacher JE Hatch EE . Why Representativeness Should Be Avoided. Int J Epidemiol (2013) 42:1012–4. 10.1093/ije/dys223 24062287 PMC3888189

[2] Ebrahim S Davey Smith G . Commentary: Should We Always Deliberately Be Non-Representative? Int J Epidemiol (2013) 42(4):1022–6. 10.1093/ije/dyt105 24062291

[3] Chiolero A . How Infodemic Intoxicates Public Health Surveillance: From a Big to a Slow Data Culture. J Epidemiol Community Health (2022) 76(6):623–5. 10.1136/jech-2021-216584 35135859

[4] Chiolero A Carmeli C . When Data Generate Populations. Int J Epidemiol (2024) 53(1):dyad166. 10.1093/ije/dyad166 38086011

[5] Degtiar I Rose S . A Review of Generalizability and Transportability. Annu Rev Stat Appl (2023) 10(1):501–24. 10.1146/annurev-statistics-042522-103837

[6] Westreich D Edwards JK Lesko CR Cole SR Stuart EA . Target Validity and the Hierarchy of Study Designs. Am J Epidemiol (2019) 188(2):438–43. 10.1093/aje/kwy228 30299451 PMC6357801

[7] Cambridge Dictionary. Representativeness. (2026). Available online at: https://dictionary.cambridge.org/dictionary/english/representativeness (Accessed February 18, 2026).

[8] Rudolph JE Zhong Y Duggal P Mehta SH Lau B . Defining Representativeness of Study Samples in Medical and Population Health Research. BMJ Med (2023) 2(1):e000399. 10.1136/bmjmed-2022-000399 37215072 PMC10193086

[9] Lundberg I Johnson R Stewart BM . What Is Your Estimand? Defining the Target Quantity Connects Statistical Evidence to Theory. Am Sociol Rev (2021) 86(3):532–65. 10.1177/00031224211004187

[10] Keiding N Louis TA . Perils and Potentials of Self-Selected Entry to Epidemiological Studies and Surveys. J R Stat Soc (2016) 179(2):319–76. 10.1111/rssa.12136

[11] Hossein MN Zuniga A Thi Nguyen N Flores H Wang J Tarkoma S Population Digital Health: Continuous Health Monitoring and Profiling at Scale. Online J Public Health Inform (2024) 16:e60261. 10.2196/60261 39565687 PMC11601140

[12] Carrière TC Boeschoten L Struminskaya B Janssen HL de Schipper NC Araujo T . Best Practices for Studies Using Digital Data Donation. Qual Quant (2025) 59(Suppl. 1):389–412. 10.1007/s11135-024-01983-x 40191694 PMC11971172

[13] Wehner MR Nead KT Linos E . Correlation Among Cancer Incidence and Mortality Rates and Internet Searches in the United States. JAMA Dermatol (2017) 153(9):911–4. 10.1001/jamadermatol.2017.1870 28658470 PMC5817428

[14] Wecker H Maier D Ziehfreund S Fox FAU Erhard I Vehreschild JJ Cancer Incidence and Digital Information Seeking in Germany: A Retrospective Observational Study. Sci Rep (2024) 14(1):10184. 10.1038/s41598-024-60267-4 38702333 PMC11068859

[15] Lazer D Kennedy R King G Vespignani A . Big Data. The Parable of Google Flu: Traps in Big Data Analysis. Science (2014) 343(6176):1203–5. 10.1126/science.1248506 24626916

[16] Pearl J . Generalizing Experimental Findings. J Causal Inference (2015) 3(2):259–66. 10.1515/jci-2015-0025

[17] Lee RS Hanage WP . Reproducibility in Science: Important or Incremental? Lancet Microbe (2020) 1(2):e59–60. 10.1016/S2666-5247(20)30028-8 32835329 PMC7255127

[18] Galea S Tracy M . Participation Rates in Epidemiologic Studies. Ann Epidemiol (2007) 17(9):643–53. 10.1016/j.annepidem.2007.03.013 17553702

[19] van Alten S Domingue BW Faul J Galama T Marees AT . Reweighting UK Biobank Corrects for Pervasive Selection Bias due to Volunteering. Int J Epidemiol (2024) 53(3):dyae054. 10.1093/ije/dyae054 38715336 PMC11076923

[20] Schoeler T Speed D Porcu E Pirastu N Pingault JB Kutalik Z . Participation Bias in the UK Biobank Distorts Genetic Associations and Downstream Analyses. Nat Hum Behav (2023) 7(7):1216–27. 10.1038/s41562-023-01579-9 37106081 PMC10365993

[21] Lesko CR Buchanan AL Westreich D Edwards JK Hudgens MG Cole SR . Generalizing Study Results: A Potential Outcomes Perspective. Epidemiology (2017) 28(4):553–61. 10.1097/EDE.0000000000000664 28346267 PMC5466356

[22] Sudlow C Gallacher J Allen N Beral V Burton P Danesh J UK Biobank: An Open Access Resource for Identifying the Causes of a Wide Range of Complex Diseases of Middle and Old Age. Plos Med (2015) 12(3):e1001779. 10.1371/journal.pmed.1001779 25826379 PMC4380465

[23] Hernán MA Hsu J Healy B . A Second Chance to Get Causal Inference Right: A Classification of Data Science Tasks. Chance (2019) 32(1):42–9. 10.1080/09332480.2019.1579578

[24] Galea S . An Argument for a Consequentialist Epidemiology. Am J Epidemiol (2013) 178(8):1185–91. 10.1093/aje/kwt172 24022890

[25] Lund JL Matthews AA . Identifying Target Populations to Align With Decision-Makers' Needs. Am J Epidemiol (2024) 193(11):1503–6. 10.1093/aje/kwae129 38897981 PMC11538562

[26] Dahabreh IJ Hernán MA . Extending Inferences From a Randomized Trial to a Target Population. Eur J Epidemiol (2019) 34(8):719–22. 10.1007/s10654-019-00533-2 31218483

[27] Stantcheva S . How to Run Surveys: A Guide to Creating Your Own Identifying Variation and Revealing the Invisible. Ann Rev Econ (2023) 15(1):205–34. 10.1146/annurev-economics-091622-010157

[28] Bradley VC Kuriwaki S Isakov M Sejdinovic D Meng XL Flaxman S . Unrepresentative Big Surveys Significantly Overestimated US Vaccine Uptake. Nature (2026) 600(7890):695–700. 10.1038/s41586-021-04198-4 34880504 PMC8653636

[29] Smith B . Generalizability in Qualitative Research: Misunderstandings, Opportunities and Recommendations for the Sport and Exercise Sciences. QRSEH (2017) 10(1):137–49. 10.1080/2159676x.2017.1393221

[30] Bell KJL Medcalf E Stanaway FF . Ensuring Target Trials and Target Estimands Are on Target for Intended Use Populations. Int J Epidemiol (2025) 54(4):dyaf138. 10.1093/ije/dyaf138 40694833

[31] Levy NS Arena PJ Jemielita T Mt-Isa S McElwee S Lenis D Use of Transportability Methods for Real-World Evidence Generation: A Review of Current Applications. J Comp Eff Res (2024) 13(11):e240064. 10.57264/cer-2024-0064 39364567 PMC11542082

[32] Vuong Q Metcalfe RK Ling A Ackerman B Inoue K Park JJ . Systematic Review of Applied Transportability and Generalizability Analyses: A Landscape Analysis. Ann Epidemiol (2025) 104:61–70. 10.1016/j.annepidem.2025.03.001 40064249

[33] Manke-Reimers F Brugger V Bärnighausen T Kohler S . When, Why and How Are Estimated Effects Transported Between Populations? A Scoping Review of Studies Applying Transportability Methods. Eur J Epidemiol (2025) 40(3):255–73. 10.1007/s10654-025-01217-w 40249515 PMC12137380

